#  Knowledge about the importance of antenatal care among females of child bearing age living in a suburban community of Lahore

**DOI:** 10.12669/pjms.35.5.1256

**Published:** 2019

**Authors:** Hina Ahmed, Iram Manzoor

**Affiliations:** 1 Dr. Hina Ahmed, MBBS, PGD Nutrition, M-Phil Community Medicine, MHPE Scholar Assistant Professor, Department of Community Health Sciences, FMH College of Medicine & Dentistry, Lahore, Pakistan; 2 Prof. Dr. Iram Manzoor, FCPS. Community Medicine MCPS Medical Education, Akther Saeed Medical and Dental College, Lahore, Pakistan

**Keywords:** Antenatal care, Anemia, Hypertension, Diabetes, Tetanus Toxoid (TT), Institutional deliveries

## Abstract

**Background and Objectives:**

During the past few decades, females had been making conscious decision to have antenatal checkup from skilled health care provider due to improved education which had played a vital role to enhance their awareness regarding the importance of this comprehensive health service. The objective was to find out the perception of females of child bearing age about the importance of antenatal care during their last pregnancy, living in a suburban community of Kot-Lakhodare Lahore.

**Methods:**

Females of reproductive age (15-49 years) living in Kot-Lakhodare were enrolled. Through a structured questionnaire, using convenient sampling technique, 1224 females of childbearing were interviewed through a cross sectional survey. The study was completed between January till August 2016. Question pertaining to their sociodemographic characteristics, perception about the importance of antenatal care services and comorbid conditions during the last pregnancy were asked. Data was analyzed by using SPSS version 21. The ethical approval both institutional and individual were duly taken.

**Results:**

Mean age was 32 ±7.8SD years with education up to primary. Three quarter of females were un- employed with monthly income less than rupees 25,000 / month. All females 869(73%) who had perception about importance of antenatal checkup during their last pregnancy had no history of anemia, hypertension, diabetes and abortion (p<0.05). These females coming for the antenatal checkup delivered uneventfully by normal vaginal route and preferred government and private hospital for delivery and were vaccinated against Tetanus Toxoid (p<0.05).

**Conclusion:**

The females of child bearing age during the last pregnancy had adequate knowledge about the importance of antenatal care which played an important role in prevention of comorbid conditions like anemia, hypertension, diabetes and risk of abortion. Moreover, they had the perception of importance of institutionalized deliveries, tetanus Toxoid vaccination coverage during pregnancy.

## INTRODUCTION

Despite major advances in health care, 830 women die every day due to pregnancy related complication according to WHO estimates.^1^ Major reasons of these maternal deaths in developing countries can be avoided if the women receive quality care during pregnancy.^1^ Antenatal care (ANC) provides comprehensive health services to the expected mother.^2^ It ensures that, any complication during pregnancy will be addressed and that is why it has been an important pillar of Safe Mother hood initiative.^2^ According to an estimate anemia, hemorrhage, hypertensive disorders gestational diabetes are some of the major causes of maternal death during pregnancy.^3^ Antenatal checkups substantiate maternal wellbeing and prevent complications by timely advice and treatment.^3^ According to the PDHS 2017-18 report, 86% of the mothers in Pakistan received antenatal care^1^ from skilled health care providers at least once for their last birth and 50% had four or more ANC visits., the ANC coverage in Punjab was, 93.3%, 55.5% in Baluchistan, 80.1% in Khyber Pakhtonkha, Sindh it was 85.7%, in ICT Islamabad was 93.6% while in FATA it was 71%.^4^

Different factors influence the awareness regarding importance of antenatal checkups which include role of education, income, support of the family and equitable distribution of health services between rural and urban population^4^. It has been documented that as the educational status of the females in urban settings is improving leading to increase in the awareness regarding importance of antenatal care.^4^ It is seen that educated mothers make conscious decision of availing ANC services from government or private hospitals.^4^ The estimates also support that, maternal deaths can also be reduced by capacity building of the health personals both at government and private sector who can identify the pregnancy related complication and at the same time provide health education to the expectant mother.^4^

Moreover, tetanus toxoid coverage in Pakistan range from 60%-74%, this low vaccination coverage is due to many factors most important among them is lack of information about its benefits among health care providers and expecting females.^4^ Media and health care providers are playing negligible role in spreading awareness. Inadequate knowledge among women of reproductive age especially in rural settings than urban which can only be improved by health education and effective communication.^5^

It is a documented fact that, antenatal checkup through quality services and practices can improve the pregnancy outcome therefore the objective was to find out whether females living in a suburban community of Kot-Lakhodare Lahore, had the perception of importance of antenatal care services during their last pregnancy which can affect the comorbid conditions during pregnancy.

## METHODS

The data was collected through a cross-sectional house hold survey conducted in community of Kot-Lakhodare, comprising an estimated population of 62,785 people. It comes under the union council 174 Wagha Town. It was a suburban community. Total 1224 married female between age of 15-49 year, living at their place of residence for at least six months were being interviewed through a questionnaire using convenient sampling technique. The participants excluded were those visiting Lakho-dare, with age less than 15 or more than 49 years, unmarried, too frail, mentally unfit to participate in the study and not willing to give informed consent. Using a structured questionnaire, the participants were asked questions pertaining to their sociodemographic profile like age, level of education, occupation, marital status, duration of marriage, employment status and house hold income. The questions were also related to maternal health including age at marriage and age at1st pregnancy, and perception about the importance of antenatal checkups during their last pregnancy and health seeking behavior. The entire data was compiled and analyzed by using software of SPSS version 21. For the quantitative variable means and standard deviations were calculated. The relationship between the categorical variables was determined by chi-square test by keeping the p value significant at less 0.05 at 95% confidence interval and power of the study was kept at 80%. The ethical approval both individual and institutional were duly taken.

## RESULTS

The mean age of the participants in years was 32 ±7.8SD. Mean age of marriage in years was 22±3.8SD. Average number of antenatal visits during last pregnancy were 3±1.4SD, Married female were 1191(97%) with 705(57.6%) had an education up to primary. Three quarters of the females were un employed 1090(89%) (Table-I).

**Table I:** Sociodemographic profile of the participants.

Sociodemographic profile	n (%)
Mean age (years)	32±7.8SD
Mean age at marriage	21±3.34SD
Mean age of 1^st^ pregnancy	22±3.8SD
Average number of antenatal visits during last pregnancy	3±1.4SD
***Marital status***	
Married	1191(97)
Separated	6(0.5)
Divorced	5(0.4)
Widowed	22(1.8)
***Education status***	
Up to Primary	705(57.6)
Up to intermediate	463(37.8)
Bachelor	56(4.6)
***Working status***	
Unemployed	1091(89)
Employed	133(11)
***Income in Rupees /month***	
≤25,000	718(80)
≥25,000	178(20)

SD: Standard Deviation

Monthly income of 781(82%) participants was less than rupees 25,000 per month (Table-I). The mean age at which they had first pregnancy 22 ±3.8SD years and 896(73%) of the females had perception about the importance of antenatal checkup during the last pregnancy. Total number of the female who had the perception about antenatal checkup during their last pregnancy had no history of anemia 492(54.9%), 627(70%) no previous abortions, 684(76.3) had no history of hypertension and 851(95) no history of diabetes (p<0.05) (Table-II). Almost two third (68%) of the women delivered their last born child through normal vaginal route (p<0.05) (Table-II) and they preferred government 414(46%) and private 377(42%) hospitals for health advice for antenatal care (p<0.05) respectively (Table-III). Females who had the perception of importance of antenatal checkup during their last pregnancy received Tetanus toxoid injection during the 7^th^ and the 8^th^ month of pregnancy (p<0.05)

**Table II:** The relationship between the knowledge of importance of antenatal checkup and medical conditions, mode and place of delivery and Tetanus Toxoid vaccination status during last pregnancy.

Variables		Importance of Antenatal check-up during last pregnancy			

		Yes n (%) 896(73)	No n (%) 328(27)	X^2^	p- value
Anemia	Yes	404(45)	66(20.1)	63.2	0.000
	No	492(54.9)	262(79.9)		
Abortion	Yes	269(30)	61(18.6)	15.9	0.000
	No	627(70)	267(81.4)		
Hypertension	Yes	212(23.7)	29(8.8)	33.3	0.000
	No	684(76.3)	299(91.2)		
Diabetes	Yes	45(5)	9(2.7)	2.95	0.05
	No	851(95)	319(97.3)		
Mode of delivery	NVD	608(68)	276(85)	33	0.000
	C-section	288(32)	50(15)		
Place of delivery	TBA at home	212(23)	192(59)	140	0.000
	Government hospital	392(44)	91(28)		
	Private hospital	292(33)	43(13)		
Vaccination against Tetanus Toxoid	Yes	682(88)	94(29)	233	0.000
	No	214(24)	234(71)		

P value <0.05 considered as significant, X^2^ chi-square measure of association.

**Table III:** Health seeking behavior of the participant for antenatal check.

	Antenatal checkup Yes n(%) 896(73)	Antenatal checkup No n(%) 328(27)	p-value
Government Hospitals	414(46)	111()	0.000
Private	377(42)	58(17)	
Homeopathy	5(0.6)	2(0.6)	
Local dais	98(10.9)	157(47.9)	
Family Planning Clinic	1(0.1)	0(0)	
Both Private And Government Hospitals	1(0.1)	0(0)	

P value <0.05 considered as significant.

## DISCUSSION

The role of antenatal check-up during pregnancy cannot be denied.^6^ It is the most beneficial comprehensive service to prevent pregnancy related complication which intern improves pregnancy outcome.^6^ In the current study 73% of the women had perception about the importance of ANC services during their last pregnancy with average three visits. Internationally, 86 per cent of pregnant women access antenatal care with a skilled health personnel at least once, only three in five (62 per cent) receive at least four antenatal visits.^6^ In regions with the highest rates of maternal mortality, such as sub-Saharan Africa and South Asia, women received at least four antenatal visits (52 per cent and 46 per cent, respectively).^5^ According to UNICEF fact sheet, ANC coverage in India was between 50-74%, Bangladesh was between 20-49% while in Nepal it was between 50-74%.^5^

The results of the current study showed that about 50% of the females of child bearing age preferred institutional delivery whether government or private. According to the PDHS 2017-18 report, 86% of the mothers in Pakistan received antenatal care^1^ from skilled health care providers at least once for their last birth and 50% had four or more ANC visits.^4^ This had shown to reduce the risk of prematurity, fetal growth retardation and fetal asphyxia.^7^ Furthermore, it had been documented in many other studies that females who were attending at least one antenatal visit before delivery had lower incidence of pregnancy related complications like hypertension, gestational diabetes and anemia.^5^,^6^ Additionally, antenatal screening had been helpful in identification of infections like malaria, tetanus, syphilis and urinary tract infections.^7^,^8^ The same had been depicted in the current study that all those females who had perception about the importance of antenatal checkup during their last pregnancy, had been significantly related to lower proportion of history of anemia, hypertension and diabetes and abortions. These finding signifies that the perception of importance of antenatal care and checkup can prevent many comorbid conditions during pregnancy.^9^,^10^

Moreover, majority of the females who had the perception of importance of antenatal checkups, had already received tetanus toxoid vaccination during their last pregnancy. Although the Tetanus Toxoid coverage in Pakistan range from 60%-74% over last decade.^11^,^12^ Moreover, the low vaccination coverage had been attributed to the fact that reproductive age females had in adequate knowledge about the benefits of vaccine which was not conveyed by the health care providers and media.^12^ It is essential to emphasize here that antenatal care services provide opportunities to the females to get them checked and helps in early identification of the pregnancy related problems.^13^,^14^ Moreover, the awareness regarding the importance of antenatal checkups and tetanus toxoid vaccination coverage can be increased by improving the education status of our females.^14^,^15^

The statistics world-wide, highlighted that mother’s educational status and income can substantially improve the antenatal check up coverage during pregnancy.^4^ According to PHDC 2017-18 report, 76% of women with no education had received ANC services during their last pregnancy, while the women with educational background of middle or higher education, 95% had antenatal care services.^4^ In the current study, majority of females had educational background of only up-to primary level, even then they had perception about the importance of antenatal care during their last pregnancy. The percentage had been found to increase with every passing year due increase in education and wealth.^6^ It is a documented fact that health education was more acceptable by the women who had some education, at the same time only an educated female could realize their reproductive needs and rights than an uneducated woman.^8^,^9^ Moreover, only an educated female could guide other females about the importance of antenatal care services and would prefer to have institutionalized delivery rather than a home delivery.^8^ The same picture had been depicted in the current study, that most of the female were delivered through normal vaginal route and they preferred government and private institution for delivery rather than home delivery.^9^,^10^ It had been seen through literature that institutionalized deliveries had played a pivotal role in reduction of maternal and infant mortality. Level of education and the consistent utilization of institutional delivery were found to be significantly associated. Educated female would always prefer institutional antenatal check -up and delivery.^12^

### Limitations of the study:

The prime limitation of this study was that it was a cross-sectional household survey and convenient sampling technique was used. In future, a survey should be designed with bigger sample size through randomization so that true prevalence can be determined.

### Recommendations

The current study was based on the perception about the importance of antenatal care services and history of comorbid condition during their last pregnancy but it had not been able to determine the evidence of anemia, hypertension and diabetes on laboratory workup. A future study is highly recommended in which the finding can be based on laboratory tests.

## CONCLUSION

The females of child bearing age during the last pregnancy had adequate knowledge about the importance of antenatal care which played an important role in prevention of comorbid conditions like anaemia, hypertension, diabetes and risk of abortion. Moreover, they had the perception of importance of institutionalized deliveries and tetanus Toxoid vaccination coverage during pregnancy.
